# Nitric Oxide is Necessary for Diazoxide Protection Against Ischemic Injury in Skeletal Muscle

**Published:** 2012

**Authors:** Hossein Farahini, Marjan Ajami, Jalaledin Mirzay Razaz, Nahid Azad, Mansooreh Soleimani, Seyyed Abdulmajid Ayatollahi, Nahid Abotaleb, Habibolah Peyrovi, Hamidreza Pazoki-Toroudi

**Affiliations:** a *Department of Orthopedic Surgery, Tehran University of Medical Sciences, Tehran, Iran.*; b *Department of Nutrition, School of Public Health, Tehran University of Medical Sciences, Tehran, Iran.*; c *Physiology Research Center, Tehran Univercity of Medical Sciences, Tehran, Iran. *; d *Department of Community Nutrition, Faculty of Nutrition and Food Technology, Shahid Beheshti University of Medical Sciences and Health Services, Tehran, Iran.*; e *Nano Medicine and Tissue Engineering Center, Shahid Beheshti University of Medical Sciences, Tehran, Iran.*; f *Cellular and Molecular Research Center, Tehran University of Medical Sciences, Tehran, Iran. *; g *Department of Pharmacognosy, School of Pharmacy, Shahid Beheshti University of Medical Sciences, Tehran, Iran.*; h *Phytochemistry Research Center, Shahid Beheshti University of Medical Sciences, Tehran, Iran.*; i *Department of Surgery, Taleghani Hospital, Shahid Beheshti University of Medical Sciences, Tehran, Iran*.; j *Nano Vichar Pharmaceutical Ltd, Tehran, Iran.*

**Keywords:** Ischemia reperfusion, Diazoxide, K_ATP_ channels, Nitric Oxide, iNOS

## Abstract

Ischemia reperfusion injury (IR injury) is a common problem in clinical conditions. Researches have frequently revealed that ATP- sensitive potassium (K_ATP_) channels and nitric oxide plays a role in protection against ischemic injury in skeletal muscle. The present study aimed at evaluating the possible link between this two pathways.

Sixty-eight male wistar rats, were pretreated with saline, diazoxide (K_ATP_ opener; 45 mg/Kg, IP), glibenclamide (K_ATP_ inhibitor; 5 mg/Kg), or L-NAME (iNOS inhibitor; 20 mg/Kg, IP) before 3 h ischemia and 2 h reperfusion. Activities of antioxidant enzymes superoxide dismutase (SOD) and catalase (CAT), and the level of malondialdehyde (MDA) and expression of iNOS were measured in muscle tissue.

Tissue MDA content was significantly increased by IR (p < 0.001). Diazoxide significantly decreased the IR-induced elevation of tissue MDA level (p < 0.05) and Glibenclamide increased MDA (p < 0.05 vs. IR group). L-NAME inhibited the effect of diazoxide on decreasing MDA (p < 0.01 vs., diazoxide+IR group) and IR decreased the activity of SOD and CAT (p < 0.01), while pretreatment with diazoxide increased activity of SOD and CAT (p < 0.01). Glibenclamide decreased SOD and CAT activity after IR (p < 0.05). L-NAME pretreatment in diazoxide-treated rats abolished the effect of diazoxide on increasing the activity of SOD and CAT (p < 0.05 vs. Diaz+IR). Expression of iNOS was increased by IR (p < 0.01 vs. Sham group). Diazoxide significantly decreased iNOS expression after IR (p < 0.05 vs. IR). L-NAME significantly decreased iNOS expression after IR (p < 0.01) in diazoxide-treated rats (p < 0.01 vs. Diaz+IR).

In conclusion, the results of present study suggested a NO dependent protective effect for diazoxide against muscle IR injury.

## Introduction

One of the common problems in clinical conditions such as infarction, stroke, myocutaneous tissue transfer, thrombolytic therapy, balloon angioplasty and cardiopulmonary bypass is ischemia reperfusion injury (IR injury) which causes tissue damage by restricting blood supply and subsequent restoration of vascular supply and production of oxygen derived free radicals (1). IR affects the antioxidant defenses in favor of the generation of reactive oxygen species (ROS) (2). It has been demonstrated that exposure of tissue to brief periods of IR can protect tissue against severe insults of IR injury, a phenomenon named ischemic preconditioning (IPC) (3).

Many studies on cardiac protection about IPC suggested that opening of ATP-sensitive potassium (K_ATP_) channels (4, 5) and presence of nitric oxide (6) are essential for beneficial effects of preconditioning. Further studies suggested that the cardioprotective effects of K_ATP_ openers are associated with mitochondrial K_ATP_ (mK_ATP_) channels activation (7). Studies on other organs demonstrated that the activation of K_ATP_ channels protected neuronal tissue and skeletal muscle which express mK_ATP_ (8, 9). The results of studies by Pang *et al *(8) confirmed the preconditioning in skeletal muscle and showed that this protective effect could be abolished by K_ATP _channels blockers such as sodium 5-hydroxydecanoate (5-HD). Specific mitochondrial mK_ATP_ channel opener diazoxide and BMS-191095 increased the ischemic tolerance in the skeletal muscle (10, 11). Other studies suggested that mK_ATP_ channels are involved both as a trigger and a mediator of hindlimb preconditioning of skeletal muscle against infarction in pigs (12).

Two general classes of nitric oxide synthases (NOS) enzymes include calcium dependent (cNOS, including the endothelial (eNOS) and neuronal (nNOS) isoforms) and a calcium-independent isoform (iNOS) (13). NO plays an important role in cardiac protection against IR injury (14). Previously it had been demonstrated that myocardial protection was lost in presence of NOS inhibitors (15) and expression of iNOS increased in cardiac tissue after IPC (16). It has also been confirmed that NOS activity is involved in mediating the protection during ischemic tolerance (17, 18). The interaction between NO-dependent pathways and mK_ATP_ channels in induction of protection against IR injury has been demonstrated in previous studies (19-21) and confirmed the activation of K_ATP_ channels by NO in cardiac tissue. The main goal of the present study is to trace the possible interaction between NO system and K_ATP _channels in protection against IR injury in skeletal muscle of rats.

## Experimental

All experimental protocols were approved by the Ethics Review Committee for Animal Experimentation of Tehran University of Medical Sciences and were in accordance with the NIH Guide for the Care and Use of Laboratory Animals.

*Animals and drugs*

A total of 64 male Wistar rats, weighing between 200 and 240 g, were used in the present study. The rats were housed in groups of eight with food and water available, under 12h light/dark cycle (light 7:00 a.m. to 7:00 p.m.) and controlled temperature (22 ± 2°C).

The following drugs were administered intraperitoneally: pentobarbital (45 mg/Kg, IP, Sigma, St. Louis, MO, USA), L-NAME (20 mg/Kg, IP; nonselective NOS inhibitor, 20 mg/Kg, Sigma), Diazoxide (40 mg/Kg, IP: K_ATP_ channels opener, Sigma), and Glibenclamide (5 mg/Kg, IP: non selective K_ATP_ channels blocker, 0.3mg/Kg, Tehrancheme, Tehran, Iran).

*Induction of Ischemia*

The rats were anesthetized with pentobarbital (45 mg/Kg, ip). An incision was made in the inner side of the hind leg from the inguinal ligament to the tendon calcaneus insertion. Then dissected femoral vessels including the artery and vein were clamped with a single clamp. The area was closed through the ischemia period. For reperfusion periods, the clamp of the femoral vessels of animals was removed and. The muscle tissues was homogenized in cold KCl solution (1.5%) to attain a 10% homogeny suspension and used for biochemical assays (22).

*Design*

The rats were separated as 8 experimental groups (Table 1). The sham control group received anesthetics similar to those of IR groups, and operated without inducing of ischemia. Diazoxide (40 mg/Kg, ip), and Glibenclamide (5 mg/Kg, ip) were injected 30 min before ischemia, while L-NAME (20 mg/Kg, ip) was injected 45 min before ischemia.

*Molecular evaluations*

The activities of antioxidant enzymes superoxide dismutase (SOD) and catalase (CAT), and the level of malondialdehyde (MDA) were measured in the supernatant obtained from centrifugation at 14,000 rpm.

The method of thiobarbituric acid was applied to determine the level of MDA. MDA, as a thiobarbituric acid reactive substance (TBARS), reacts with thiobarbituric acid (TBA) to produce a red-colored complex with maximal absorbance of 532 nm. The MDA concentration was calculated from the intensity of pink color of the final product at 532 nm. Results were expressed as nmol MDA per g of wet tissue.

Catalase (CAT) and Superoxide Dismutase (SOD) activities were determined using the commercial kits [Superoxide Dismutase Activity Colorimetric Assay Kit (abcam, ab65354) and Catalase Assay Kit (abcam, ab83464)]. CAT activity was measured by a spectrophotometric assay of hydrogen peroxide based on formation of its yellow stable complex with ammonium molybdate. SOD activity was determined by using xanthine oxidase method based on O2•− generation. The SOD and CAT activities were expressed as units per mg tissue protein (U/mg protein) in tissue samples.

*iNOS expression in muscle tissue*

iNOS expression in tissue was determined by western blotting. Briefly, the cell protein was extracted from 100 mg of tissue in western blot lysis buffer and the samples were centrifuged at 22,000 g for 20 min (4°C). Extracted protein (100 μg) was mixed with sample buffer and boiled for 5 min. Then samples were separated on a 7% gel and electroblotted to a nitrocellulose membrane for 2 h. Membranes were blocked overnight and incubated with rabbit anti-iNOS (Santa Cruz Biotechnology, CA) followed by incubation with horseradish peroxidase conjugated secondary antibody for 1 h at room temperature. The bands were finally visualized with the ECL chemiluminescence system (Amersham) and the film was developed and used for measurement of optical density.

*Statistical analysis*

Since the data showed normal distribution pattern using Kolmogorov-Smirnov test as well as homogeneity of variance, the results were statistically evaluated by One-Way Analysis of Variance (ANOVA) and post-hoc Tukey’s test. All data were expressed as Mean ± Standard Error of Mean (SEM). Statistical significance level was determind as p < 0.05.

## Results and Discussion

All animals used in the present study remained alive towards the end.

*Muscle tissue MDA after IR*

**Figure 1:**
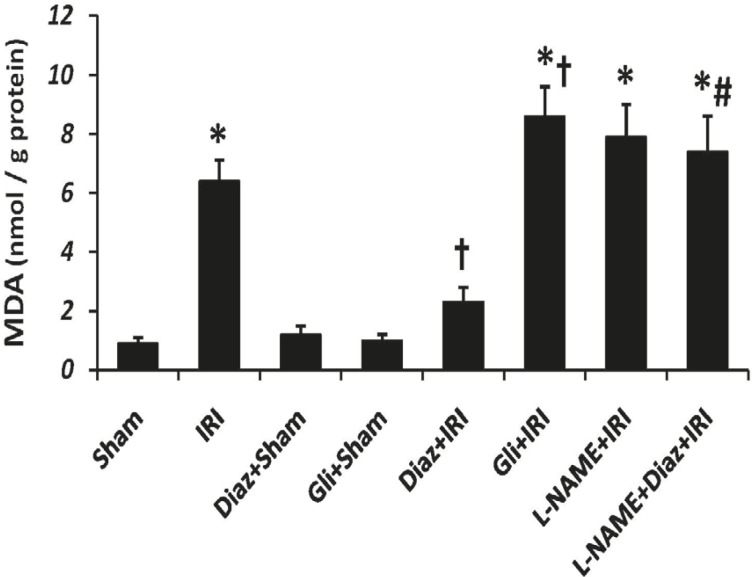
Muscle tissue Malondialdehyde (MDA) level as an index of lipid peroxidation was measured 2 h after the reperfusion. Data are given as Mean ± SEM. Ischemia reperfusion injury (IRI), Diaz: Diazoxide (40 mg/Kg), Gli: Glibenclamide (5 mg/Kg), L-NAME (20 mg/Kg). * p < 0.001 vs. Sham, † p < 0.05 vs. IRI and # p < 0.01 vs. Diaz+IRI group

Tissue MDA content was significantly increased by IR (6.4 ± 0.7 nmol/g protein, p < 0.001, Figure 1); however, diazoxide significantly decreased the IR-induced elevation of tissue MDA level (2.3 ± 0.5 nmol/g protein, p < 0.05, Figure 1). Glibenclamide significantly increased tissue MDA content after IR (p < 0.05 vs. IR group). MDA contents were not changed by L-NAME after IR. However, MDA contents of muscle tissue in diazoxide-treated rats which had been pretreated with L-NAME was significantly more than diazoxide+IR group (p < 0.01 vs., Figure 1).

*CAT and SOD activity*

**Figure 2:**
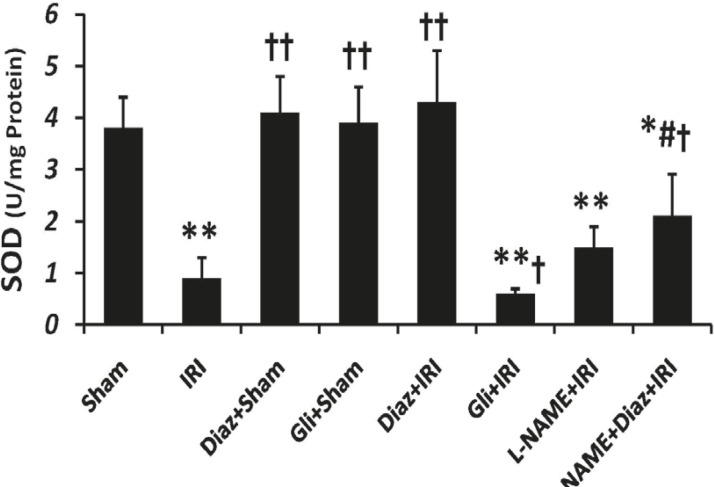
Effect of treatment with diazoxide and L-NAME on activity of antioxidant enzyme SOD in tissue samples prepared from hind limb muscle. Data are expressed as Mean ± SEM in all groups. Ischemia reperfusion injury (IRI), Diaz: Diazoxide (40 mg/ Kg), Gli: Glibenclamide (5 mg/Kg), L-NAME (20 mg/Kg). * p < 0.05 and ** p < 0.01 vs. Sham, †p < 0.05 and ††p < 0.01 vs. IRI, and # p < 0.05 vs. Diaz+IRI group

**Figure 3:**
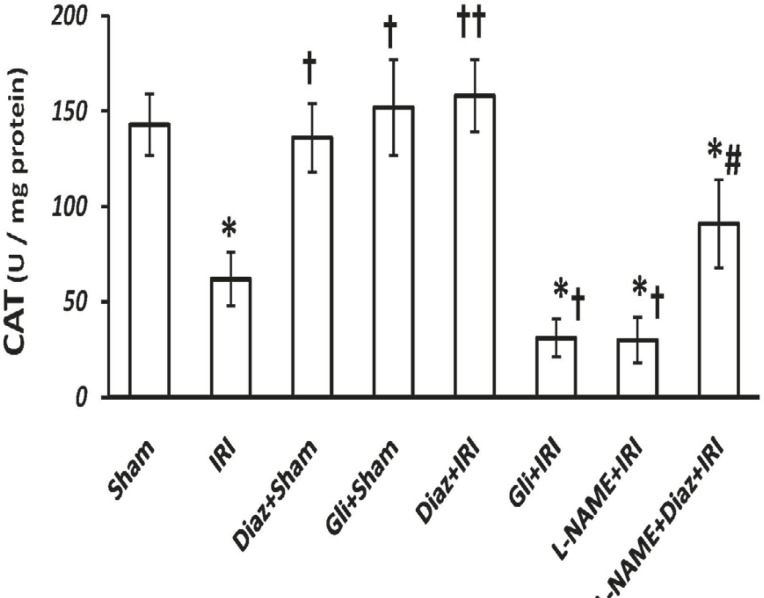
Effect of treatment with diazoxide and L-NAME on activity of antioxidant enzyme CAT in tissue samples prepared from hind limb muscle. Data are expressed as mean ± SEM in all groups. Ischemia reperfusion injury (IRI), Diaz: Diazoxide (40 mg/Kg), Gli: Glibenclamide (5 mg/Kg), L-NAME (20 mg/Kg). * p < 0.01 vs. Sham, † p < 0.05 and †† p < 0.01 vs. IRI, and # p < 0.05 vs. Diaz+IRI group

IR decreased the activity of SOD and CAT in muscle tissue from (3.8 ± 0.6 and 143 ± 16 U/g protein; respectively) to (0.9 ± 0.4 and 62 ± 14 U/g protein, p < 0.01; respectively). Pretreatment with diazoxide 40 mg/Kg in sham-operated animals had no effect on SOD and CAT activity, while it increased the activity of SOD and CAT (4.3 ± 1 and 158 ± 19, p < 0.01, respectively; Figures 2 and 3). Glibenclamide had no effect on SOD and CAT activity of sham-operated animals, but decreased SOD and CAT activity after IR injury (p < 0.05 vs. IR group). 

Pretreatment with L-NAME had no significant effect on tissue SOD activity, but the activity of CAT decreased significantly in L-NAME treated rats compared with IR group (p < 0.05). L-NAME pretreatment in diazoxide-treated rats abolished the effect of diazoxide on increasing the activity of SOD and CAT (2.11 ± 0.8 and 91 ± 23, p < 0.05 vs. Diaz+IR rats, respectively). 

*iNOS expression*

**Figure 4:**
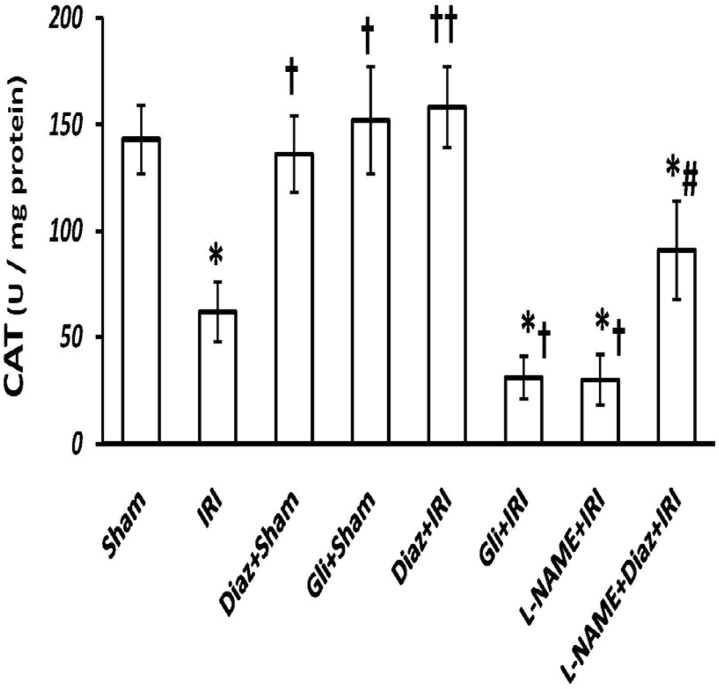
iNOS protein expression was measured in muscle tissue 2 h after reperfusion period in different groups. The Ratio of iNOS expression in each group in respect to the sham group was normalized and the results are shown as means ± SEM in graph of Upper panel (A). The results of Western blot analysis for iNOS protein levels illustrated in Lower panel (B). Ischemia reperfusion injury (IRI), Diaz: Diazoxide (40 mg/Kg), Gli: Glibenclamide (5 mg/Kg), L-NAME (20 mg/Kg). *p < 0.05 and **p < 0.01 vs. Sham, † p < 0.01 and †† p < 0.001 vs. IRI, and ## p < 0.01 vs. Diaz+IRI group

Expression of iNOS was increased by IR (p < 0.01 vs. Sham group, Figure 4). Glibenclamide and diazoxide had no effect on expression of iNOS in sham operated animals. Diazoxide significantly decreased iNOS expression after IR (p < 0.05 vs. IR). L-NAME significantly decreased iNOS expression after IR (p < 0.001 vs. IR). Expression of iNOS also decreased significantly in diazoxide treated rats which had received L-NAME before (p < 0.01 vs. Diaz+IR group, Figure 4).

The main purpose of the present work was to evaluate the role of NO in the protective pathway of K_ATP_ channels. Glibenclamide, the blocker of K_ATP_ channels, increased tissue damage resulted from IR and Diazoxide, an opener of K_ATP_ channels, protected muscle tissue against IR injury as shown through decreased MDA level and increased SOD and CAT activity. To evaluate the role of NO, L-NAME was applied to block NO K_ATP_ channels were first identified in cardiac muscle (23) nevertheless, it was then revealed that it is also present in other tissues, including smooth production. L-NAME treatment decreased iNOS expression and abolished the effects of diazoxide on decreasing lipid peroxidation and increasing antioxidant enzymes. muscles (24) and skeletal muscles (25). These channels are inhibited by ATP and stimulated by ADP in normal conditions. Recent studies have also suggested that K_ATP _channel openers mimic the effects of ischemic preconditioning by interacting with K_ATP_ channels in the inner mitochondrial membrane (26).

The role of K_ATP_ channels opening in induction of tolerance against IR injury of skeletal muscle is confirmed in the present study. Diazoxide is a selective mitochondria K_ATP_ channel opener, which has been reported to preserve the microvascular integrity of IR injured tissues. The effect of diazoxide on skeletal muscle IR injury has been evaluated in study by Wei *et al*, on cremaster muscles and manifested that diazoxide reduced the number of rolling, adhering, and transmigrating leukocytes, while these effects were blocked by chelerythrine (protein kinase C inhibitor) and demonstrated a PKC-dependent pathway for diazoxide protection against IR injury (27). It seems that diazoxide reduces the excess production of ROS by mitochondria on reperfusion period in mitochondria and prevents from cell apoptosis (28). In present study, reduced levels of MDA and increased antioxidant enzymes activity confirmed the protective effects of diazoxide in molecular level.

IR injury distorts the balance between vasoconstricting factors (ROS, thromboxane A, and endothelin) and vasodilator factors (NO) (29, 30), and causes to marked vasospasm in the muscle arteries during early reperfusion after prolonged warm ischemia (31). In spite of the considerable evidences on the role of NO in the etiology of IR injury, the results of studies on NO are paradoxical, where low doses of NO were found to be protective and high doses harmful (32). In our study, iNOS expression increased after IR injury, suggesting that NO is participates in IR-induced injury. Increased expression of iNOS has revealed large amounts of NO production which is converted to peroxynitrite and other reactive products, leading ultimately to tissue injury (33).

However, pretreatment with L-NAME abolished protective effects of diazoxide against skeletal muscle IR injury and suggested NO dependent pathway for K_TAP_ channels’ opener. The expression of iNOS in diazoxide treated groups decreased significantly in comparison with the IR injury group, but it was also significantly more than sham group. Such amount of iNOS expression is enough to supply the NO required for diazoxide-induced protection while complete blocking of iNOS and NO production by L-NAME removed required NO from tissue and abolished diazoxide protection. The link between K_ATP_ channels and NO in preconditioning of cardiac tissue has been demonstrated previously (19-21). Since L-NAME can inhibit other isoforms of NOS, therefore the inhibitory effect of L-NAME on diazoxide induced protection may relate to its inhibitory effects on other NOS isoforms such as eNOS. eNOS-derived NO has reduced the IR-induced expression of intercellular adhesion molecule-1 (ICAM-1) and vascular cell adhesion molecule-1 (VCAM-1) and reduced neutrophil adhesion and margination and tissue damage (34). Further evaluations are required to precisely determine which NOS isoform is involved in diazoxide-induced protection.

In conclusion, the results of present study suggested that the effects of diazoxide on muscle tissue protection against IR injury is NO dependent and confirmed the interaction between NO and K_ATP_ channel pathways.
